# The core triacylglycerol toolbox in woody oil plants reveals targets for oil production bioengineering

**DOI:** 10.3389/fpls.2023.1170723

**Published:** 2023-04-03

**Authors:** Yunpeng Cao, Qiang Li, Lin Zhang

**Affiliations:** ^1^ School of Health and Nursing, Wuchang University of Technology, Wuhan, China; ^2^ Wuhan Botanical Garden, Chinese Academy of Sciences, Wuhan, China; ^3^ College of Forestry, Central South University of Forestry and Technology, Changsha, Hunan, China; ^4^ College of Basic Medical Sciences, Hubei University of Chinese Medicine, Wuhan, China

**Keywords:** woody oil plants, lipid biosynthesis, triacylglycerol pathway, seeds, expression

## Abstract

Woody oil plants are the most productive oil-bearing species that produce seeds with high levels of valuable triacylglycerols (TAGs). TAGs and their derivatives are the raw materials for many macromolecular bio-based products, such as nylon precursors, and biomass-based diesel. Here, we identified 280 genes encoding seven distinct classes of enzymes (i.e., G3PAT, LPAAT, PAP, DGAT, PDCT, PDAT, and CPT) involved in TAGs-biosynthesis. Several multigene families are expanded by large-scale duplication events, such as *G3PATs*, and *PAPs*. RNA-seq was used to survey the expression profiles of these TAG pathway-related genes in different tissues or development, indicating functional redundancy for some duplicated genes originated from the large-scale duplication events, and neo-functionalization or sub-functionalization for some of them. Sixty-two genes showed strong, preferential expression during the period of rapid seed lipid synthesis, suggesting that their might represented the core TAG-toolbox. We also revealed for the first time that there is no PDCT pathway in *Vernicia fordii* and *Xanthoceras sorbifolium*. The identification of key genes involved in lipid biosynthesis will be the foundation to plan strategies to develop woody oil plant varieties with enhanced processing properties and high oil content.

## 1 Introduction

Triacylglycerols (TAGs) are the primary form of energy storage in most living organisms (Gibbons et al., 2000; Durrett et al., 2008). They accumulate in plant seeds and provide nutrients and energy for subsequent germination and seedling development (Yang and Benning, 2018). These storage lipids are mainly composed of polysaturated fatty acids (Mu and Porsgaard, 2005; Lieb et al., 2019), which have great nutraceutical and nutritional value (Lung and Weselake, 2006; Zhang et al., 2019). Therefore, they are important precursors for chemical industry products, also providing sources for human nutrition (Lung and Weselake, 2006; Liu et al., 2011). At present, TAGs are also increasingly becoming important raw materials for the production of nylon precursors, biofuels, lubricants, detergents, and paints, as well as biomass diesel (Durrett et al., 2008; Barreira et al., 2009; Nabizadeh et al., 2011; Xu and Shanklin, 2016).

TAGs exist in all eukaryotes, such as protists, fungi, animals, and plants (Coleman and Lee, 2004). As a common metabolic pathway, the biosynthesis of TAGs seems to be conserved from bacteria to plants and humans (Yang et al., 2012). The biosynthesis of TAG mainly occurs in two different cell compartments, so it can be divided into two independent sets of reactions (Buchanan et al., 2015). In the first step, the fatty acids, or acyl, chains of TAG are primarily produced from precursor carbon molecules in the plastid. These compounds are then transported to the endoplasmic reticulum (ER), where they act as precursors for membrane and storage lipids production. In general, the TAG assembly sequentially consumes acyl-CoA using substrate glycerol-3-phosphate (G3P) with diverse enzymes. The phosphatidic acids and lysophosphatidic acids catalyzed by lysophosphatidic acid acyltransferase (LPAAT) and Glycerol-3-phosphoglycerol acyltransferase (G3PAT) were produced, respectively (Takeuchi and Reue, 2009; Athenstaedt, 2021). After the removal of phosphate, phosphatidic acids are converted to diacylglycerols, the precursor of triacylglycerols. The 3-sn-phosphatidate phosphohydrolase or phosphatidic acid phosphohydrolase (PAP) catalyzes the dephosphorylation of phosphatidic acid to produce diacylglycerols, which is the precursor of TAGs (Neumann and Römheld, 1999; Nakamura et al., 2007). Both phospholipid: diacylglycerol acyltransferase (PDAT) and acyl-CoA: diacylglycerol acyltransferase (DGAT) as acyl donors, convert diacylglycerols (DAGs) to TAGs (Stobart et al., 1997; Dahlqvist et al., 2000). Finally, the oil bodies are produced from oleosins which are assembled by TAGs in plant seeds (Kocsis and Weselake, 1996).

It is known that the global oil reserves in oilfield reservoirs will be exhausted in the next 40 to 50 years (Wenrui et al., 2013), so there is an urgent need to find renewable alternative fuels. Biodiesel is considered to be a way to solve this problem and enters the field of scientific research because it can be made from a variety of raw materials, such as microalgae, animal fat, and plant oil (Demirbas and Demirbas, 2011; Hsieh et al., 2012; Rodionova et al., 2017; Chen et al., 2018). Among the various lipids synthesized by plant cells, TAGs have the highest biofuel value. Woody oil plants, including purpleblow maple (*Acer truncatum*), walnut (*Juglans regia*), physic nut (*Jatropha curcas*), tung tree (*Vernicia fordii*), oil palm (*Elaeis guineensis*), yellowhorn (*Xanthoceras sorbifolium*), and olive tree (*Olea europaea*), are most popular in a variety of applications, such as bioremediation, cosmetics, and biofuel. In the present study, we report the identification of the genes putatively involved in TAGs biosynthesis in woody oil plants. Subsequently, we carried out the expression patterns of these genes in different tissues and/or seed development, and then discussed the functional roles of these different gene members in various enzyme classes. Our data provide the basis for further understanding the TAGs biosynthesis and will ultimately allow the validation of biotechnological strategies to design of future studies involving manipulation of oil production in woody oil plants.

## 2 Materials and methods

### 2.1 In silico identification of genes encoding enzymes from the TAGs biosynthesis pathway

We analyzed and collected the *A. thaliana* enzymes for each of seven gene families of the TAG-pathway from Plant Metabolic Network (http://www.plantcyc.org) and downloaded the TAIR database (https://www.arabidopsis.org/). The protein sequences of purpleblow maple (*A. truncatum*), physic nut (*J. curcas*), walnut (*J. regia*), yellowhorn (*X. sorbifolium*), tung tree (*V. fordii*), oil palm (*E. guineensis*), and olive tree (*O. europaea*) were obtained from https://doi.org/10.6084/m9.figshare.12986237.v2, https://plantgenomics.snu.ac.kr, http://dendrome.ucdavis.edu/ftp/Genome_Data/genome/Reju/, http://www.gigadb.org, https://bigd.big.ac.cn/gsa/, http://genomsawit.mpob.gov.my, and http://www.gigadb.org, respectively. Subsequently, these *A. thaliana* enzymes were examined by InterProScan (http://www.ebi.ac.uk/interpro/) (Jones et al., 2014) and PFAM (http://pfam.xfam.org/) (Mistry et al., 2021). Finally, we used the BLAST and HMM model searches to identify the TAG-pathway enzymes by scanning these seven woody oil plants genome database, including purpleblow maple (*A. truncatum*) (Ma et al., 2020), walnut (*J. regia*) (Martínez-García et al., 2016), yellowhorn (*X. sorbifolium*) (Bi et al., 2019; Liang et al., 2019), physic nut (*J. curca*s) (Ha et al., 2019), tung tree (*V. fordii*) (Zhang et al., 2019), oil palm (*E. guineensis*) (Singh et al., 2013), and olive tree (*O. europaea*) (Unver et al., 2017). The inclusion criteria of putative TAG-pathway enzymes required that they have more than 50% amino acid identity with the Arabidopsis query and contain corresponding domains using PFAM (Mistry et al., 2021) and InterProScan (Mistry et al., 2021).

### 2.2 Phylogenetic and sequence analyses

MAFFT software was used to align the deduced amino acid sequences from identified enzymes (Katoh et al., 2019). Available literature data were used to perform the characteristic signatures of enzyme classes and in silico characterization of enzyme domains (Schläpfer et al., 2017). TmPred server was used to predict the transmembrane domain. Dendrograms were drawn using IQ-tree with Maximum likelihood (ML) method according to the best model as implemented in IQ-tree (Minh et al., 2020).

### 2.3 RNA‐seq expression analysis

Transcriptome or RNA-seq data for different tissues from woody oil plants were obtained and downloaded from the NCBI SRA database with accession numbers: PRJNA318350, PRJNA483508, PRJNA643637, PRJNA399212, PRJNA557096, PRJNA590386, and PRJNA445068. The fastp (https://github.com/OpenGene/fastp) was used to perform the quality-based trimming. The obtained clean reads were mapped to the corresponding genomes using the HISAT2 with default parameters. The absolute transcription abundance values of all identified TAG biosynthesis genes were estimated from fragments per kilobase million (FPKM) values obtained by StringTiec (Pertea et al., 2016; Jiang et al., 2022). The expression level values used in our study are the log2-transformed FPKM values, as described in previously published papers (Carocha et al., 2015; Cao et al., 2019; Cao et al., 2022).

## 3 Result and discussion

### 3.1 In silico identification of TAG genes encoding enzymes

**Figure 1:**
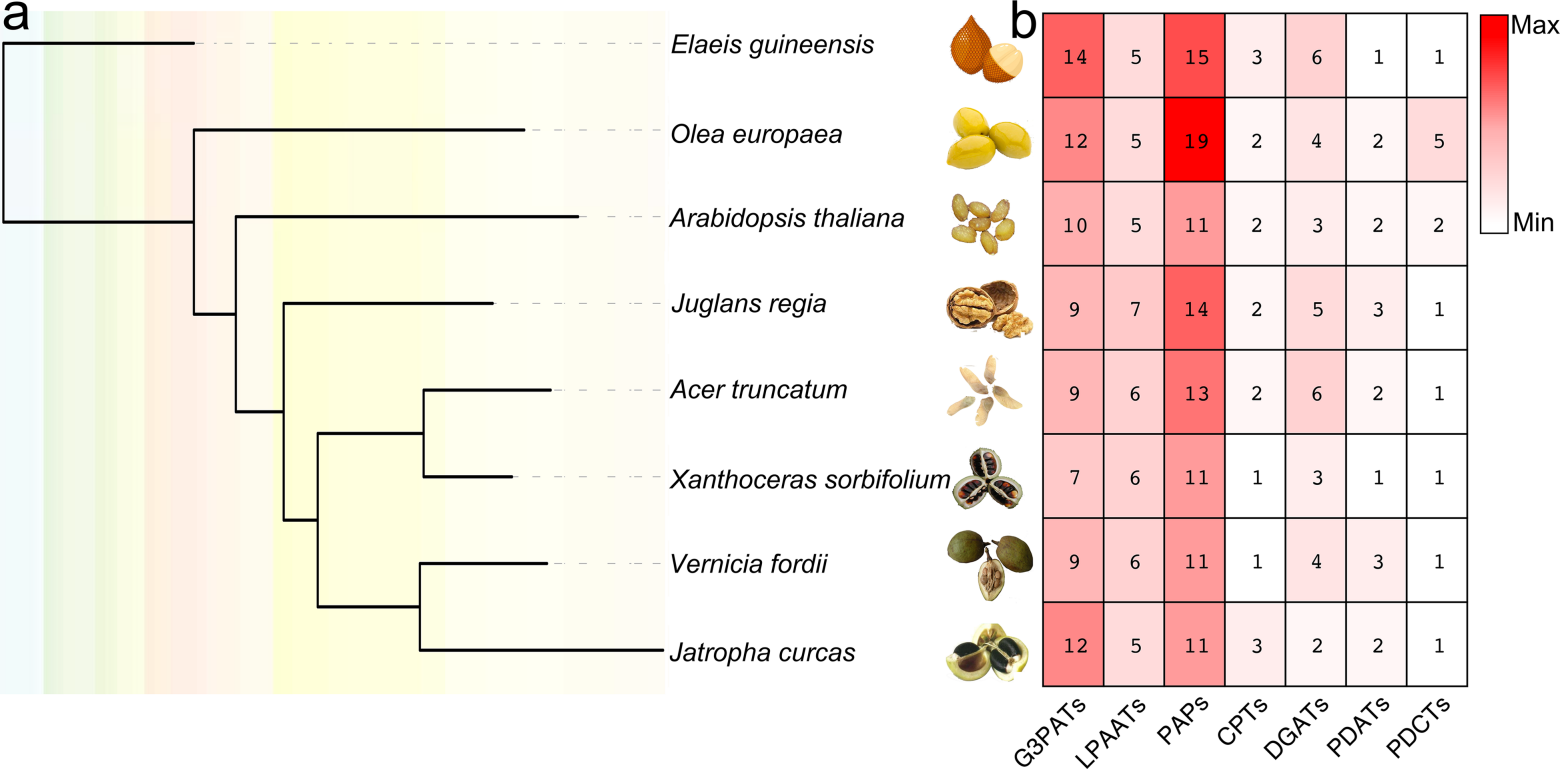
Species tree of eight plant genomes and TAG-related biosynthesis gene numbers of each species. **(A)** one-to-one orthologs were detected between genomes by Orthofinder (Emms and Kelly, 2015), and species tree was constructed by IQ-tree (Nguyen et al., 2015). **(B)** the numbers of TAG-related biosynthesis genes in each species were listed accordingly.

In the present study, both HMM model and BLAST searches were used to identify genes of seven different metabolic lipid families involved in TAG biosynthesis using known lipid enzymes as queries. The steps for gene identification contained: (1) *A. thaliana* enzymes were examined by PFAM to identify HMM model, which can scan the putative TAG-pathway enzymes; (2) BLASTp was used to further identify the putative TAG-pathway enzymes, only enzymes that have more than 50% amino acid identity with these enzymes scanned in *A. thaliana* were used as queries; (3) amino acid sequence alignment was used to identify regions of homology between putative TAG-pathway enzymes, so as to finally determine the characteristics and motifs of each type of enzyme. Finally, 280 TAG genes encoding enzymes were identified and used for further analyses (**Figure 1**, **Tables S1**, **S2**).

### 3.2 Glycerol-3-phosphate-1-acyltransferase

**Figure 2:**
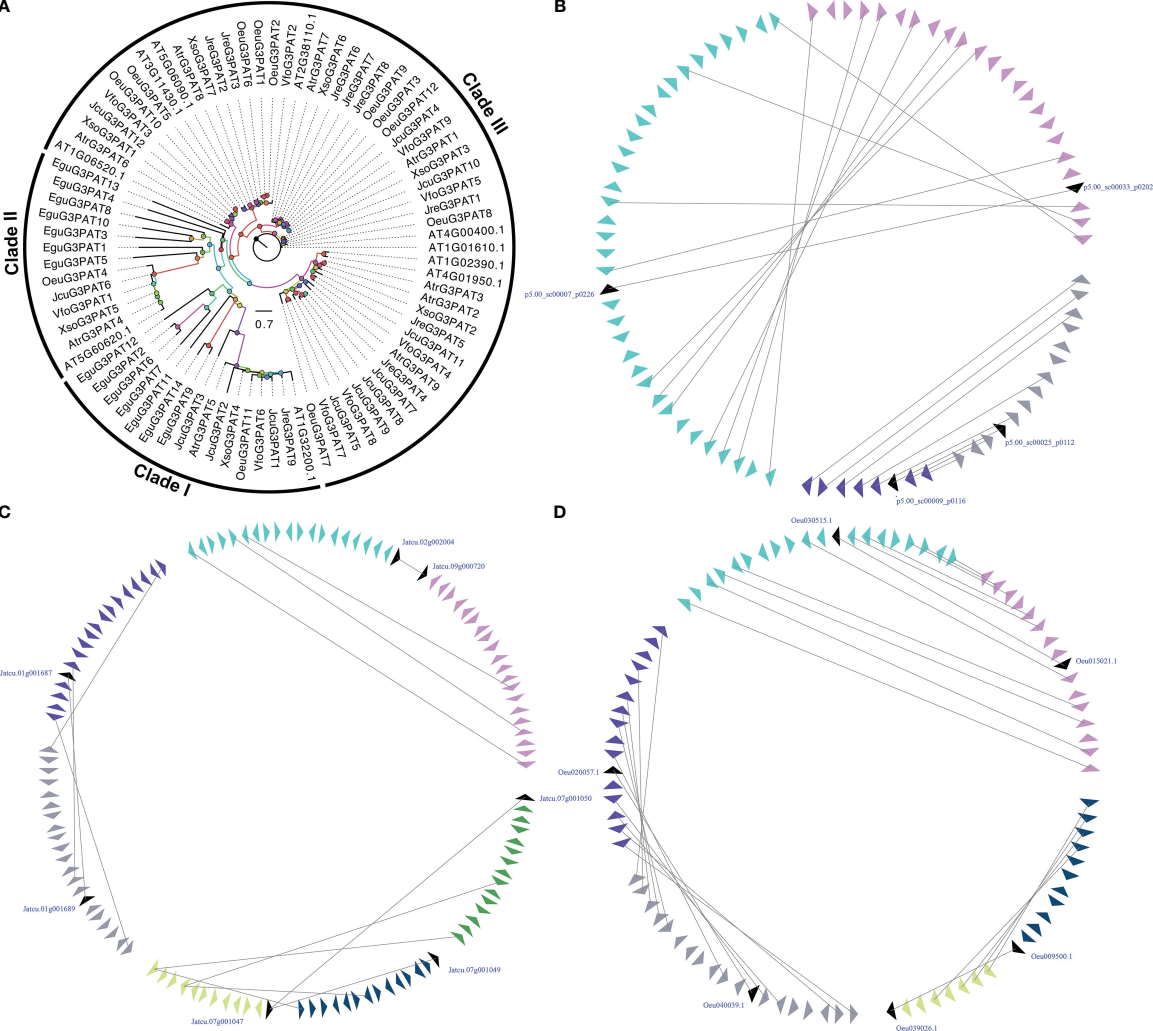
The phylogenetic tree **(A)** and microsynteny related to *G3PAT* genes in **(B)** *E guineensis*; **(C)** *O. europaea*; and **(D)** *J curcas*. The triangles indicated a gene in a family and its flanking genes, as well as the gene’s orientation. The homologous genes were connected by gray lines between two fragments.

G3PAT is the first enzyme in the Kennedy Pathway that catalyzes acyl-CoAs onto the C1 hydroxyl group of glycerol-3-phosphate or the attachment of an acyl group from either acyl-carrier proteins (acyl-ACPs) to give 1-acylglycerol-3-phosphate (Zheng et al., 2003). As early as 60 years ago, researchers have characterized the biochemical functions of G3PATs from plant and animal tissues (Kornberg and Pricer, 1953; Weiss et al., 1960). In plants, the activity of G3PATs was observed in three different plant subcellular compartments, including plastid, endoplasmic reticulum (ER), and mitochondria (Gidda et al., 2009). Seventy-two G3PATs were detected by BLASTP and HMM model with reported *A. thaliana* AtG3PATs as queries in seven woody oil plant reference genomes. The ML tree was divided these G3PATs into three clades, which was consistent with previously published manuscripts (Waschburger et al., 2018; Wang et al., 2020). The large-scale duplication events were found in the *EguG3PATs*, *OeuG3PATs*, and *JcuG3PATs*, resulting in more *G3PAT* family members than the other four woody oil plants (**Figure 2**).

The functions of most *A. thaliana AtG3PAT* genes have been characterized and identified in detail. However, the functional mechanism of woody oil plants *G3PAT* genes has not been uncovered so far, especially their role in seed oil biosynthesis. In the present study, expression profiling of *G3PATs* in different tissues or development exhibited that the transcripts accumulated in tissue-specific patterns (**Figure S1**). For example, some *G3PATs* demonstrated specific transcript accumulations in leaf, root, flower, stem, and seeds, such as *JcuG3PAT8* in stem, *JreG3PAT1* in root, and *OeuG3PAT10* in flower. The expression patterns of duplicated *G3PAT* genes exhibited distinct tissue-specific, implying that these *G3PATs* were derived from the large-scale duplication events followed by functional divergence. To further determine *G3PAT* genes that might contribute to the seed oil biosynthesis, we carried out the absolute expression level of *G3PATs* in seeds. Compared to other *G3PATs*, *AtrG3PAT1* from *A. truncatum* (2-fold higher), *XsoG3PAT2*, *XsoG3PAT3* and *XsoG3PAT4* from *X. sorbifolium* (100-fold higher), *JcuG3PAT1*, *JcuG3PAT3* and *JcuG3PAT6* from *J. curcas* (5-fold higher), *JreG3PAT1* and *JreG3PAT9* from *J. regia* (100-fold higher), *OeuG3PAT3*, *OeuG3PAT4* and *OeuG3PAT8* from *O. europaea* (20-fold higher), and *VfoG3PAT3*, *VfoG3PAT4* and *VfoG3PAT6* from *V. fordii* (3-fold higher) were relatively highly expressed in seeds, indicating these *G3PATs* might be involved in seed oil biosynthesis (**Figure S1**).

### 3.3 Lysophosphatidic acid acyltransferase

**Figure 3:**
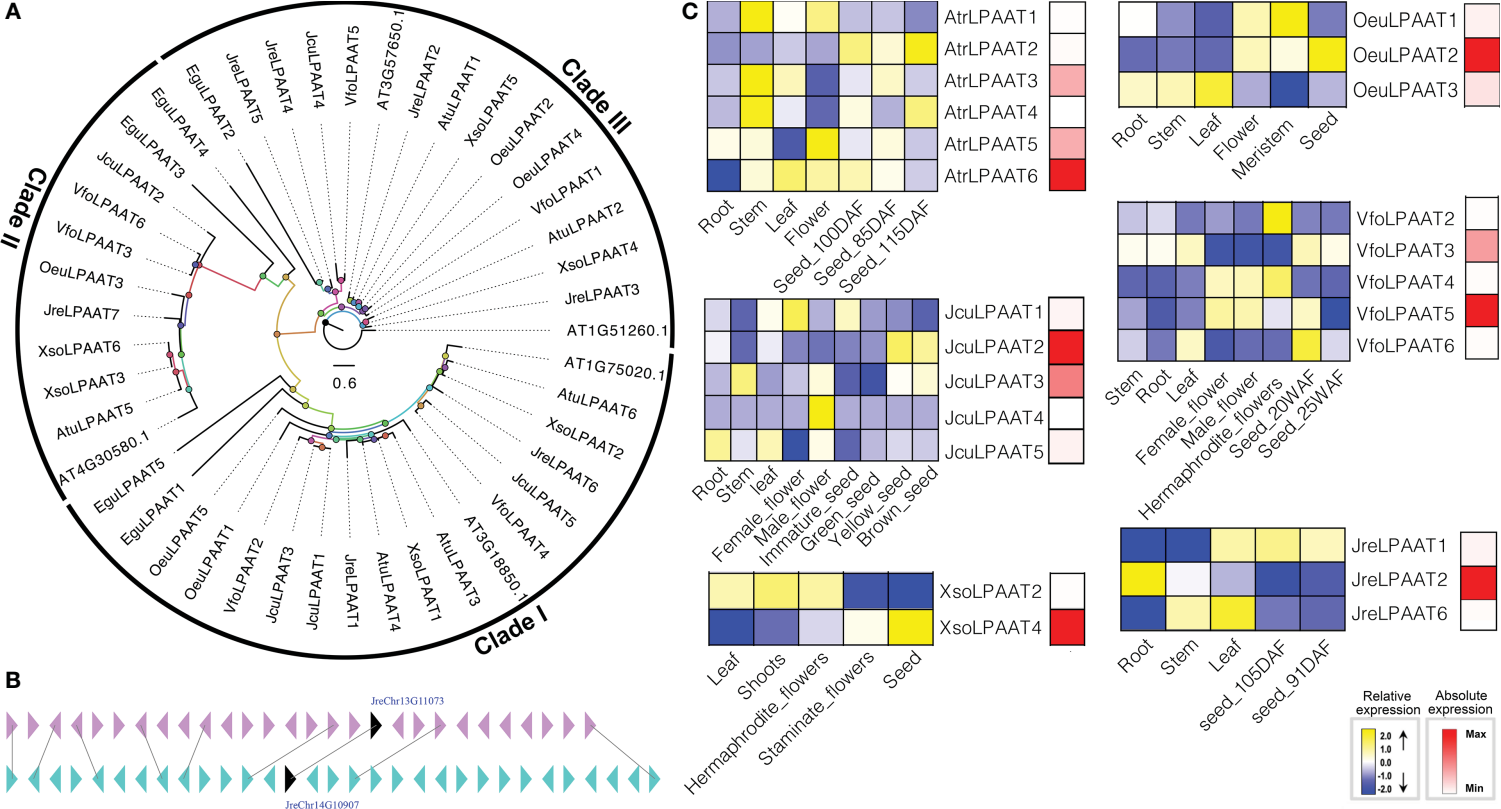
The *LPAAT* subfamily: phylogenetic tree **(A)**, microsynteny **(B)**, and expression profiles **(C)**. The triangles indicated a gene in a family and its flanking genes, as well as the gene’s orientation. The homologous genes were connected by gray lines between two fragments. The expression profiles were obtained by RNA-Seq. Blue and yellow colors indicated low-expression and high-expression, respectively. Red color suggested absolute high-expression.

LPAATs perform essential cellular functions by controlling the conversion of 1-acyl-sn-G3P (LPA) to phosphatidic acid (PA) (Hills and Roscoe, 2006). LPAAT was first characterized biochemically about 20 years ago, and then many homologous of LPAATs were identified in plants and animals (Yu et al., 2004; Körbes et al., 2016; Wang et al., 2017). In plants, there are several different isoforms of LPAAT, so their activity is related to a variety of membrane systems, such as ER, chloroplasts, and outer membrane of mitochondria (Bourgis et al., 1999; Yu et al., 2004). The *LPAAT* is an ancient and very larger gene family. The first *LPAAT* gene was cloned with containing activities about 20 years ago, then homologous of *LPAATs* have been found and characterized in many plants (Ichihara et al., 1987; Körbes et al., 2016; Bradley and Duncan, 2018; Meesapyodsuk et al., 2021). To identify the gene encoding LPAAT, we used both HMM model and BLAST to search the local genome database using proteins of *A. thaliana At4g30580, At3g57650, At1g51260, At3g18850*, and *At1g75020* as the query sequences. Finally, we identified forty *LPAATs*, including 5 *EguLPAATs* in *E. guineensis*, 5 *AtrLPAATs* in *A. truncatum*, 7 *JreLPAATs* in *J. regia*, 5 *OeuLPAATs* in *O. europaea*, 6 *VfoLPAATs* in *V. fordii*, 6 *XsoLPAATs* in *X. sorbifolium* and 5 *JcuLPAATs* in *J. curcas*, respectively. Among them, *JreLPAAT3* and *JreLPAAT5* were located in chromosome 13 and 15 and evolved from a same ancestor after undergoing large-scale duplication events (**Figure 3**).

The biological functions of *LPAATs* have been proven to be diverse (Bradley and Duncan, 2018). For example, the role of the same enzyme in affecting the downstream lipid biosynthesis pathways was significantly diverse in different tissues, highlighting the importance of tissue-specific research (Bradley and Duncan, 2018; Fahs et al., 2019). In the present study, the expression of *JreLPAAT3, JreLPAAT4, JreLPAAT5* and *JreLPAAT7, OeuLPAAT5, VfoLPAAT1, XsoLPAAT1, XsoLPAAT3, XsoLPAAT5*, and *XsoLPAAT6* were not detected in any tissues or seed development. However, the expression patterns of other *LPAAT* genes exhibited different tissue-specific, and some of them showed specific expression in seeds, which indicated their potential functions in seed oil biosynthesis. The absolute expression levels of *LPAATs* were performed to investigate which *LPAAT* might contribute to seed oil synthesis. Finally, seven *LPAATs*, including *AtrLPAAT6, JcuLPAAT2, JcuLPAAT3, JreLPAAT2, OeuLPAAT2, VfoLPAAT5*, and *XsoLPAAT4*, were determined to be involved in the biosynthesis of seed oil (**Figure 3**).

### 3.4 Phosphatidic acid phosphatase

**Figure 4:**
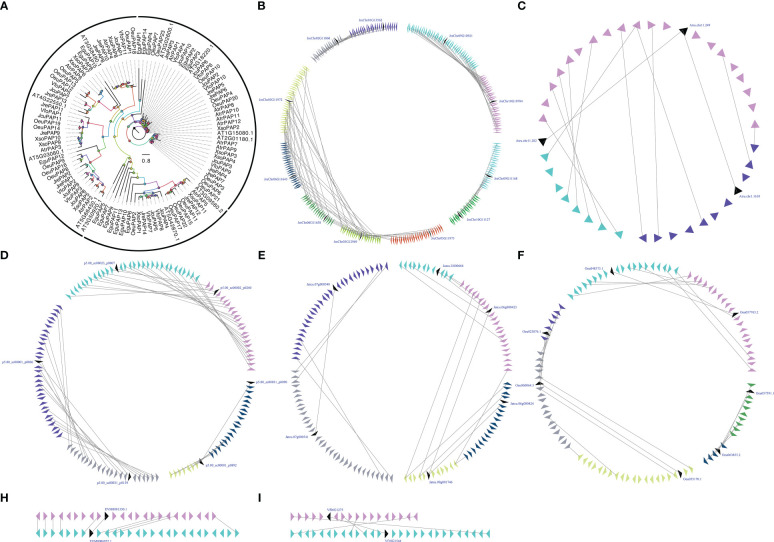
The phylogenetic tree **(A)** and microsynteny related to *PAP* genes in **(B)** *J regia*; **(C)** *A truncatum*; **(D)** *E guineensis*; **(E)** *J curcas*; **(F)** *O. europaea*; **(H)** *X. sorbifolium*; and **(I)** *V. fordii*. The triangles indicated a gene in a family and its flanking genes, as well as the gene’s orientation. The homologous genes were connected by gray lines between two fragments.

PAP is the three enzyme in the Kennedy Pathway that performs essential cellular function by controlling the conversion of PA to inorganic phosphate and sn-1,2-diacylglycerol (DAG) (Neumann and Römheld, 1999; Nakamura et al., 2007). PAP is related to the inner membrane of ER, cytoplasm, and chloroplast, its intracellular translocation may play an important role in both intracellular signaling and lipid metabolism mechanisms (Kocsis and Weselake, 1996; Perry et al., 1999; Carman and Henry, 2007). PAP activity located in the inner membrane of the plastid envelopes of *Pisum sativum* and *Spinacia oleracea* is believed to be associated with the prokaryotic glycolipid synthesis pathway, while the activity related to the microsome-associated fractions of *Persea Americana* and *Carthamus tinctorius* is believed to be related to the so-called eukaryotic lipid biosynthesis pathway (França et al., 2008). Using eleven *A. thaliana AtPAPs* (*At1g15080, At3g02600, At2g01180, At3g09560, At5g42870, At3g18220, At3g50920, At3g58490, At4g22550, At5g03080* and *At5g66450*) as BLAST queries, we identified 94 *PAPs* in seven woody oil plants. The phylogenetic analysis of PAPs from seven woody oil plants and *A. thaliana* resulted in the formation of an ML tree with three different clades for Clade I, Clade II, and Clade III. Each of the seven woody oil plants and *A. thaliana* contributed at least one PAP to these three clades, we can infer that woody oil plants *PAP* genes shared a common ancestor. To determine whether the *PAPs* have undergone the large-scale duplication events, we analyzed the relationship of the *PAPs* in these seven woody oil plants and identified two, three, four, nine, and four collinear gene pairs in *A. truncatum*, *E. guineensis*, *J. curcas*, *J. regia*, and *O. europaea*, respectively, while only one and one collinear gene pair in *X. sorbifolium* and *V. fordii*, respectively (**Figure 4**). These results might have resulted from ancient processes during the long evolutionary period (**Figure 4**).

Gene expression patterns can provide clues to gene function (Cao et al., 2020; Cao et al., 2021). To further understand the potential functions of PAPs, the RNA-seq sets were used to determine the expression patterns of these genes in different tissues. With respect to *JrePAP11, VfoPAP2, VfoPAP3, VfoPAP6, OeuPAP10, OeuPAP13, OeuPAP16, OeuPAP17, OeuPAP18, OeuPAP19, OeuPAP20, OeuPAP21, OeuPAP22, OeuPAP23, XsoPAP2, XsoPAP3, XsoPAP4, XsoPAP10*, and *XsoPAP11*, we were unable to detect any expression in tested different tissues. The involvement of PAPs in other cellular processes or the induction of other biotic and abiotic stresses might explain their undetectable expression in these tested tissues. For instance, PAPs are not only involved in glycerolipid biosynthesis, but also play important roles in the turnover and degradation of phospholipids (Cagliari et al., 2010). The expression patterns of duplicated *PAP* pairs showed different tissue-specific, suggesting that neo-functionalization or sub-functionalization occurred after the large-scale duplication events (**Figure S2**). The absolute expression levels of *PAPs* were carried out to survey which *PAP* might be involved in seed oil biosynthesis. Finally, twelve *PAPs*, including *VfoPAP5, VfoPAP9, VfoPAP10, XsoPAP1, AtrPAP4, AtrPAP10, JcuPAP2, JcuPAP3, OeuPAP1, OeuPAP3, JrePAP3*, and *JrePAP10*, were determined to be involved in the biosynthesis of seed oil (**Figure S2**).

### 3.5 Diacylglycerol acyltransferase

DGAT is a transmembrane enzyme that plays an essential role in the final rate-limiting step of TAG biosynthesis. As a key enzyme for biotechnology purposes, DGAT has great prospects for being used to increase the oil content of oil crops (Slabas et al., 2001; Lung and Weselake, 2006; Xu et al., 2008; Liu et al., 2020; Gao et al., 2021). Overexpression of DGAT in *A. thaliana* can significantly increase seed oil content (Jako et al., 2001). The substrate preference of DGAT mainly depends on the temperature, acyl-CoA concentration, and the acyl composition of the diacylglycerol (DAG) pool (Lung and Weselake, 2006). *A. thaliana* contains three genes (*At3g51520*, *At2g19450*, and *At1g48300*) that encode enzymes with DGAT activity. Through HMM model and BLAST searches, six, six, five, four, four, three and two DGATs were identified in *E. guineensis*, *A. truncatum*, *J. regia*, *O. europaea*, *V. fordii*, *X. sorbifolium* and *J. curcas*, respectively (**Figure 1**).

**Figure 5:**
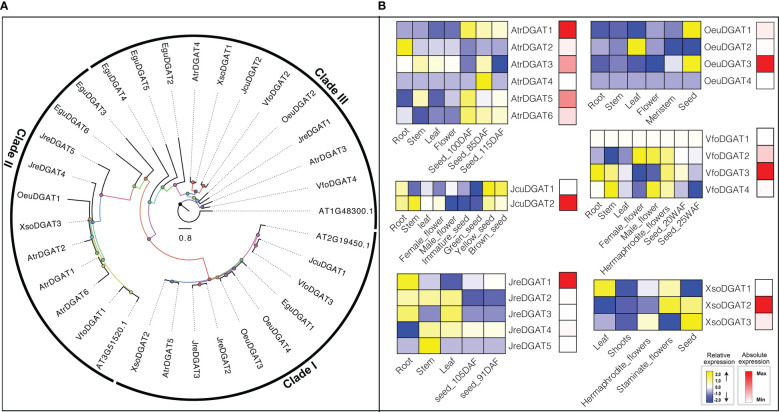
The *DGAT* subfamily: phylogenetic tree **(A)**, and expression profiles **(B)**. The expression profiles were obtained by RNA-Seq. Blue and yellow colors indicated low-expression and high-expression, respectively. Red color suggested absolute high-expression.

Different types of DGAT enzymes with different membrane-bound polypeptides have been identified in several species (Cases et al., 1998; Cases et al., 2001; Bhatt-Wessel et al., 2018; Chitraju et al., 2019), which is consistent with our results that *DGAT* genes were divided into three clades by ML tree (**Figure 5**). The roles of different types of DGAT enzymes can be species-dependent in oil production (Shockey et al., 2006). In our study, we investigated the expression patterns of *DGATs* in different tissues or development. In *O. europaea*, *OeuDGAT1* and *OeuDGAT3* were preferentially expressed seeds. Additionally, *OeuDGAT3* exhibited eightfold higher expression than *OeuDGAT1* in seeds tissues, which may consistent with its contributed to seed oil biosynthesis. Similarly, *AtrDGAT1, AtrDGAT3, AtrDGAT5, JcuDGAT2, JreDGAT1, VfoDGAT3*, and *XsoDGAT2* were identified that probably involved in seed oil biosynthesis (**Figure 5**).

### 3.6 Phospholipid diacylglycerol acyltransferase

**Figure 6:**
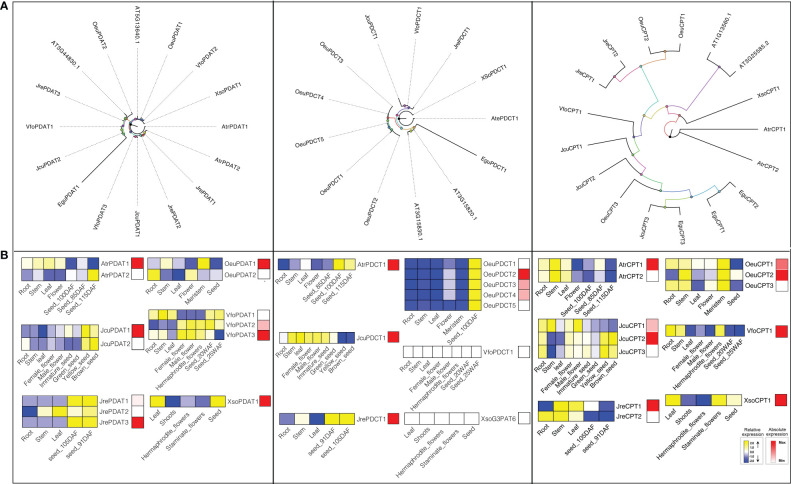
The *PDAT*, *PDCT*, and *CPT* subfamilies: phylogenetic trees **(A)**, and expression profiles **(B)**. The expression profiles were obtained by RNA-Seq. Blue and yellow colors indicated low-expression and high-expression, respectively. Red color suggested absolute high-expression.

Phospholipid diacylglycerol acyltransferase (PDAT) utilizes an acyl-CoA independent mechanism to transfer a fatty acid from the sn-2 position of phosphatidylcholine (PC) to -diacylglyerol (DAG) forming TAG and a lysophospholipid (Dahlqvist et al., 2000). Therefore, PDAT can contribute to the high levels of polyunsaturated fatty acid (PUFA) in seed oils of *A. thaliana *(Fan et al., 2013), flax (*Linum usitatissimum*) (Wickramarathna et al., 2015), and *Camelina sativa *(Marmon et al., 2017). *A. thaliana* has two *PDATs* (*At3g44830* and *At5g13640*). To detect the gene encoding PDAT in seven woody oil plants, both HMM model and BLAST were used to search the local genome databases. Finally, fourteen PDATs were identified, including 1 in *E. guineensis*, 2 in *J. curcas*, 2 in *O. europaea*, 3 in *J. regia*, 2 in *A. truncatum*, 1 in *X. sorbifolium*, and 3 in *V. fordii*, respectively (**Figure 6**).

To further understand the potential function of *PDAT* genes, we analyzed the expression profile of this gene family. The expression patterns suggested that *VfoPDAT3* was highly expressed in seed at 20 WAF (Seed oil was synthesized in large quantities during this period) compared to *VfoPDAT2* (2-fold higher) and *VfoPDAT1* (50-fold higher), indicating *VfoPDAT3* might involve the oil biosynthesis of *V. fordii* seed. Similarly, *AtrPDAT1, JcuPDAT1, JrePDAT3, OeuPDAT1, VfoPDAT3*, and *XsoPDAT1* probably involved in seed oil biosynthesis (**Figure 6**).

### 3.7 Phosphatidylcholine diacylglycerol cholinephosphotransferase

Phosphatidylcholine diacylglycerol cholinephosphotransferase (PDCT) is an enzyme that can catalyze the reversible transfer of the phosphocholine headgroup from PUFA-enriched PC to PUFA-enriched DAG (Bates and Browse, 2011). The acyl groups on DAG entering PC are driven by the action of PDCT, which are then returned to DAG after modification (Cahoon et al., 1999), suggesting that PDCT might contribute to the biosynthesis of TAG and determine the seed oil fatty acids composition (Wickramarathna et al., 2015). To determine the gene encoding PDCT, we used both HMM model and BLAST to search the local genome database using proteins of *A. thaliana At3g15830* and *At3g15820* as the query sequences. Consequently, eleven candidate *PDCTs* were identified from the oil woody plant genome databases, namely *AtrPDCT1*, *EguPDCT1*, *JcuPDCT1*, *JrePDCT1*, *VfoPDCT1*, *XSoPDCT1*, and *OeuPDCT1-5* (**Table S1**, **Figure 6**).

*E. guineensis*, *J. curcas*, *J. regia*, *A. truncatum*, *X. sorbifolium*, and *V. fordii* contained single copy PDCT, while the *O. europaea* genome had five members. *OeuPDCT1/OeuPDCT2* and *OeuPDCT3/OeuPDCT4/OeuPDCT5* were located in chromosome 10 and 21, respectively, which were derived from the lineage-specific tandem duplication followed by functional redundancy (**Table S1**, **Figure 6**). For example, the expression profiles of these five *OeuPDCTs* were very similar and were weakly expressed root, stem, leaf and meristem, and highly expressed seeds (**Figure 6**). Compared to the other four *PDCTs*, *OeuPDCT2* was highly expressed in seed (2.2-fold higher), indicating that this gene was probably involved in seed oil biosynthesis of *O. europaea*. Remarkably, the expression of *VfoPDCT1* and *XsoPDCT1* were not detected in different tissues, suggesting the PDCT pathway might not be involved in the seed oil biosynthesis of *X. sorbifolium* and *V. fordii*. Taken together, our results suggested that *AtrPDCT1*, *EguPDCT1*, *JcuPDCT1*, *JrePDCT1*, and *OeuPDCT2* might contribute to seed oil biosynthesis (**Figure 6**).

### 3.8 Choline phosphotransferase

CPT or aminoalcohol phosphotransferase (AAPT) is a key enzyme in oilseed metabolism, which can promote the TAG biosynthesis through the reversible conversion of phosphatidylcholine (PC) to diacylglycerol (DAG) (Voelker and Kinney, 2001). *A. thaliana* contains two genes (*At1g13560* and *At3g25585*) that encode enzymes with both CDP-ethanolamine: diacylglycerol choline phosphotransferase (EPT) activity and CPT activity (Liu et al., 2015b). By using *A. thaliana* CPT enzymes as queries, we have identified three, three, three, two, two, one, and one *CPTs* in *E. guineensis*, *J. curcas*, *O. europaea*, *J. regia*, *A. truncatum*, *X. sorbifolium*, and *V. fordii*, respectively. Previous studies have shown that CPT proteins from *Brassica rapa*, yeast, soybean, *A. thaliana*, and *Ricinus communis* are highly conserved, that is, they generally contain a highly conserved CDP-alcohol phosphotransferase group sequences: DGxxARxxxxxxxxGxxxDxxxD (Mcmaster and Bell, 1997; Williams and Mcmaster, 1998; Goode and Dewey, 1999; Cagliari et al., 2010). This motif was also highly conserved in CPTs among these seven woody oil plants (**Table S2**).

To further understand how *CPTs* evolved, the gene duplication events of this gene family were investigated in woody oil plants. *JcuCPT2/JcuCPT3* were located in chromosome 11, which were derived from the lineage-specific tandem duplication followed by functional redundancy (**Table S1**). For example, the expression profile of *JcuCPT2/JcuCPT3* exhibited a strong and preferential expression in seed development (**Figure 6**). The absolute expression levels suggested that *JcuCPT2* and to a lesser extent *JcuCPT1* are probably involved in seed oil biosynthesis in *J. curcas*. Similarly, *AtrCPT1, JreCPT1, VfoCPT1, XsoCPT1, OeuCPT1*, and *OeuCPT2* were also probably involved in seed oil biosynthesis (**Figure 6**).

### 3.9 Hypothetical pathways involved in the biosynthesis of lipid in woody oil plants

**Figure 7:**
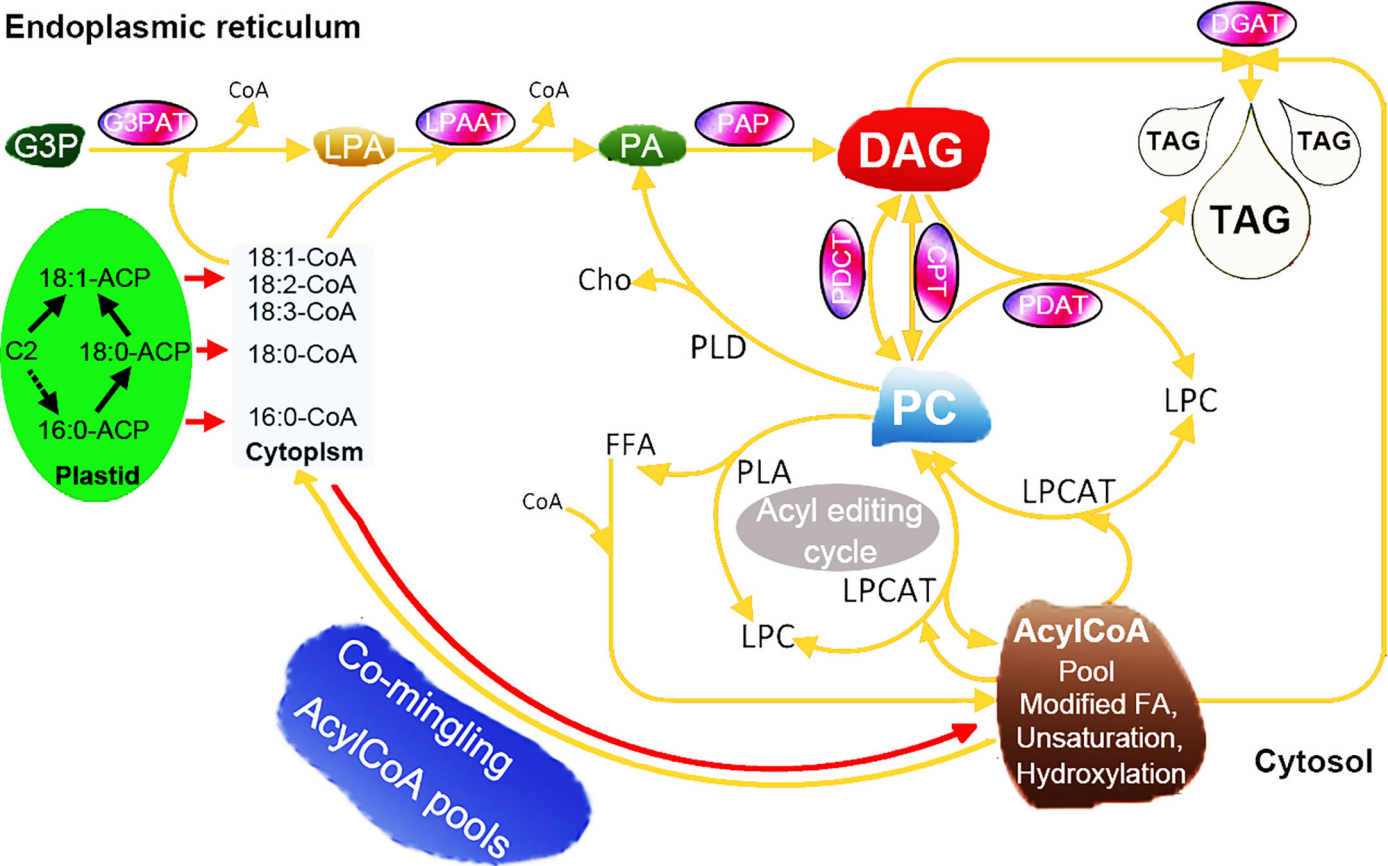
Hypothetical pathways involved in the TAG biosynthesis in seeds of woody oil plants. The TAG biosynthesis pathway was adapted from previous papers (Coleman and Lee, 2004; Cagliari et al., 2011; Bates, 2012; Parajuli et al., 2020).

The improvement and modification of seed oil composition can help to produce the production of industrially and nutritionally desirable oils (Kocsis and Weselake, 1996). To achieve this goal, it is necessary to manipulate the biosynthesis of fatty acids and TAGs in such a way that the specific fatty acids are synthesized at high rates and then effectively inserted into each position of TAGs (Yu et al., 2004). Although it is now possible to obtain genes encoding suitable fatty acid modifying enzymes from the model species *A. thaliana* and other species, the expression of these genes in transgenic plants does not produce high yields of the required oil components, making progress limited (Yu et al., 2004; Burgal et al., 2008). The biosynthesis of TAG requires the participation of multiple enzymes and the synergy of regulatory factors (Cagliari et al., 2010). Overexpression of one gene from the TAG synthesis pathway has a limited effect on increasing the oil content of seeds (Elhiti et al., 2012; Shi et al., 2012). Recently, some researchers have found that co-expression of multiple genes in transgenic lines can significantly increase the oil content. For example, Liu et al., (2015a) overexpressed *ScLPAAT, BnDGAT, BnGPAT*, and *BnGPDH* genes in *Brassica napus*, and found that the seed oil content of the overexpression lines was significantly higher than that of the control lines (Liu et al., 2015a). Here, our data highlighted 62 genes encompassing seven enzyme families involved in the core TAG biosynthesis of seeds (**Figure 7**), such as *XsoCPT1*, *AtrPDCT1*, and *VfoPDAT3*. To gain understand the expression profiles of strongly expressed and/or preferentially expressed genes, a unique expression pattern was also observed by using RNA-seq. Additionally, some other genes, such as *OeuG3PAT10* and *AtrPAP13*, were expressed in these tested tissues, indicating that these genes might be involved in other biosynthesis other than the TAG pathway.

## 4 Conclusion

Seeds of woody oil plants have been used for producing biofuels, biomass diesel, paints, and edible oil. In this study, we provide key information regarding core TAG biosynthesis, which has been limited to date. Although further studies encompassing enzymatic assays and posttranscriptional analyses are needed to determine the activity of the enzymes encoded by these genes from the TAG biosynthesis. However, our data exhibited here may help identify potential targets for future biotechnological approaches that may play crucial roles in the development of genetic strategies to control lipid biosynthesis.

## Data availability statement

The original contributions presented in the study are included in the article/**Supplementary Material**. Further inquiries can be directed to the corresponding authors.

## Author contributions

YC designed this research and then wrote the manuscript. YC, QL, and LZ participated in the evaluation of the manuscript revision. YC contributed to the provided guidance of the whole study. All authors contributed to the article and approved the submitted version.

## Supplementary material

Supplementary Table 1 Identification of TAG-related biosynthesis genes in woody oil plants

Supplementary Table 2 Amino acid sequence of TAG-related biosynthesis genes in woody oil plants

Supplementary Figure 1 The expression profiles of *G3PAT* genes in woody oil plants. The expression profiles were obtained by RNA-Seq. Blue and yellow colors indicated low-expression and high-expression, respectively. Red color suggested absolute high-expression.

Supplementary Figure 2 The expression profiles of *PAP* genes in woody oil plants. The expression profiles were obtained by RNA-Seq. Blue and yellow colors indicated low-expression and high-expression, respectively. Red color suggested absolute high-expression.
