# Supplementary figures and images for: The core triacylglycerol toolbox in woody oil plants reveals targets for oil production bioengineering
Source: Front Plant Sci. 2023 Apr 3;14:1170723. doi: 10.3389/fpls.2023.1170723 (PMC10106636; doi:10.3389/fpls.2023.1170723)

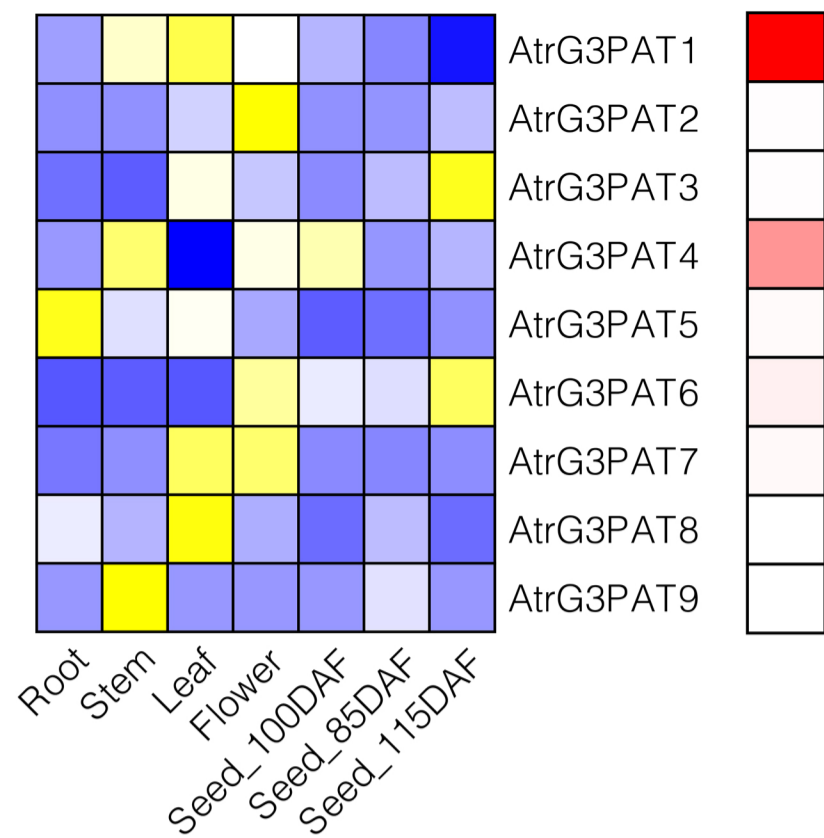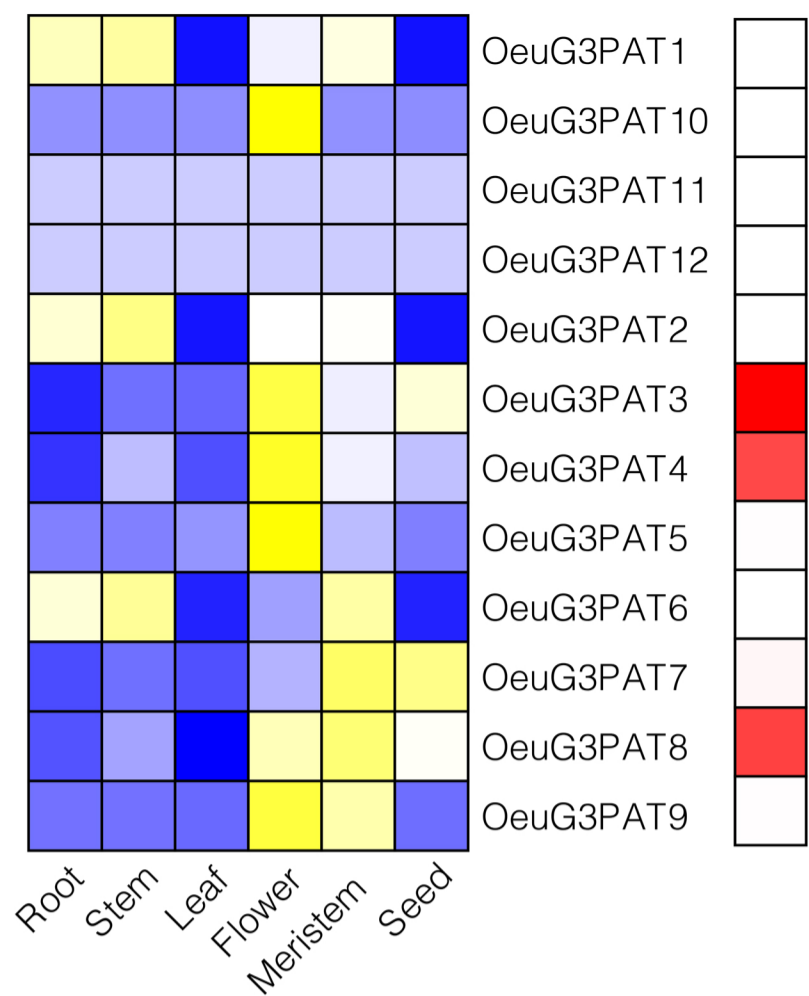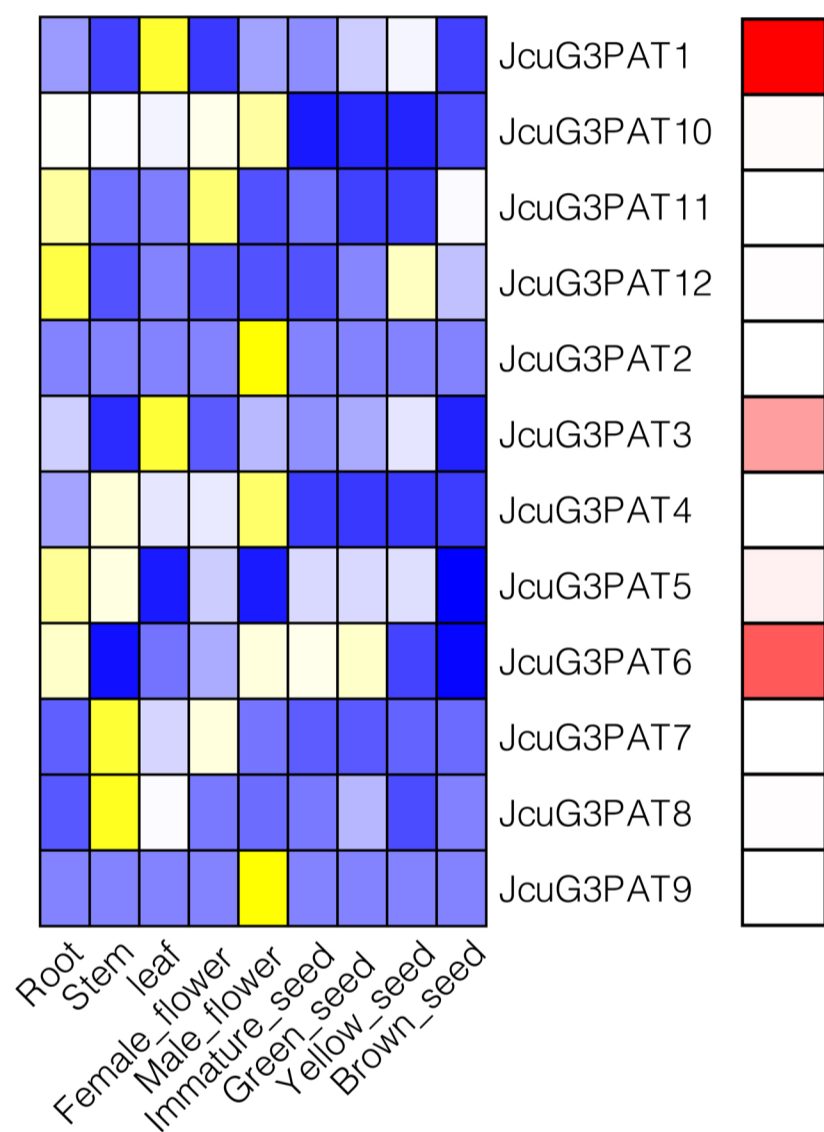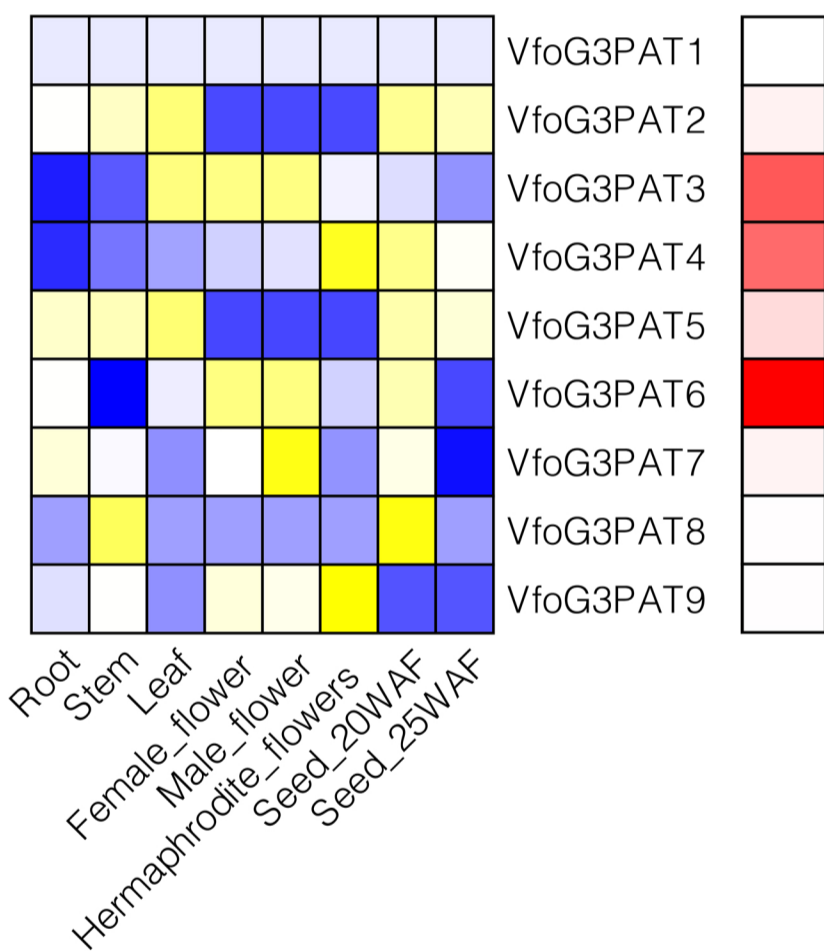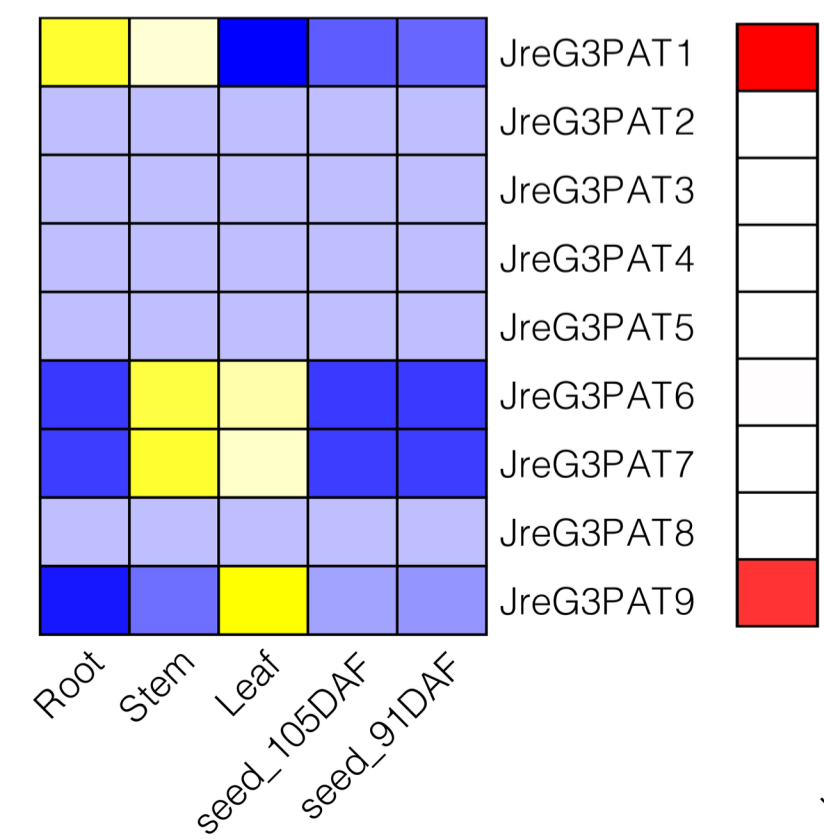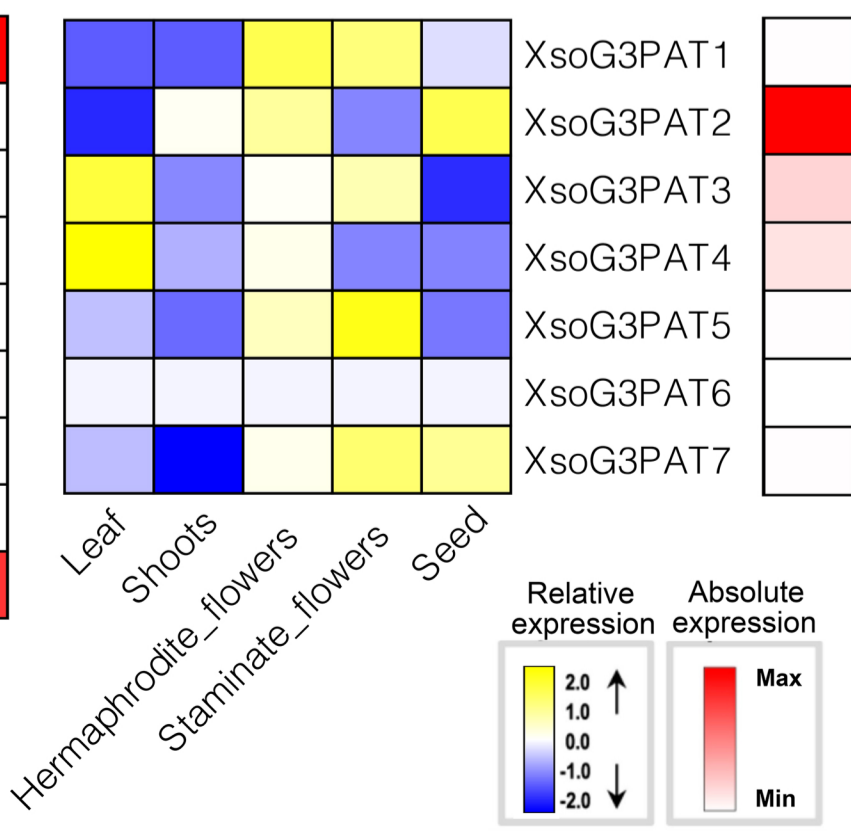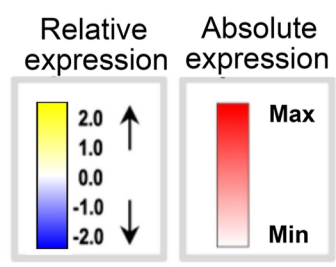

Supplement: Supplementary Figure 1 — The expression profiles of G3PAT genes in woody oil plants. The expression profiles were obtained by RNA-Seq. Blue and yellow colors indicated low-expression and high-expression, respectively. Red color suggested absolute high-expression. [file DataSheet_1.pdf]

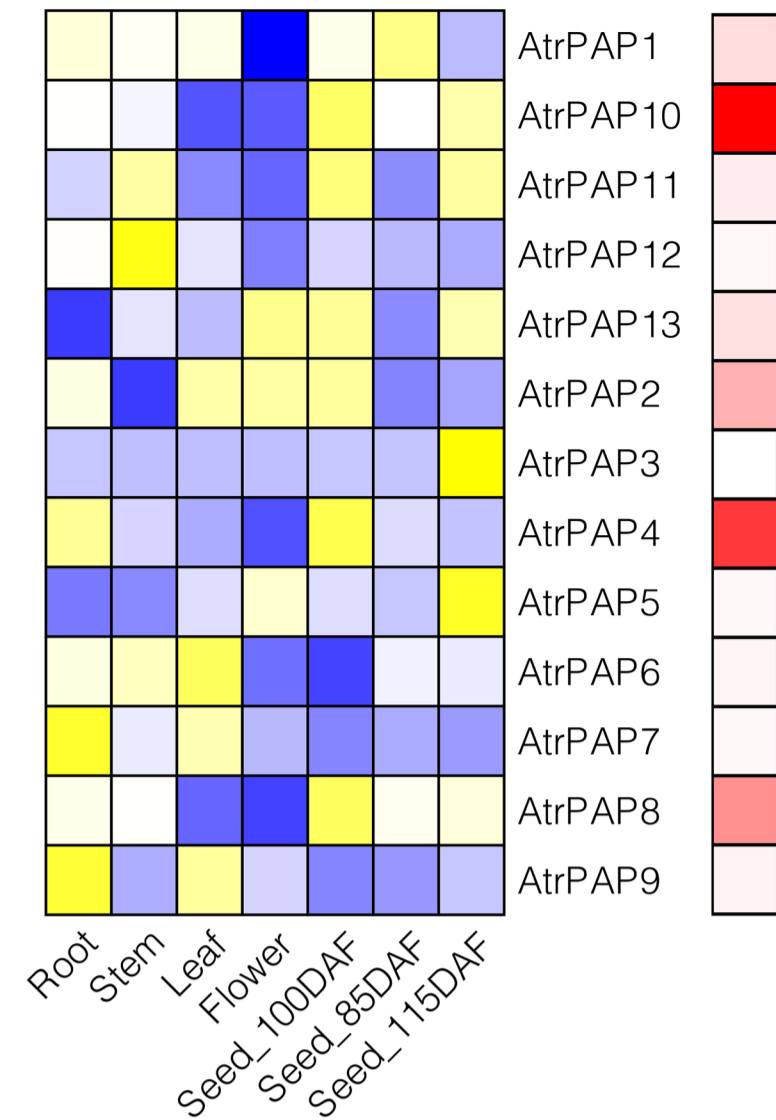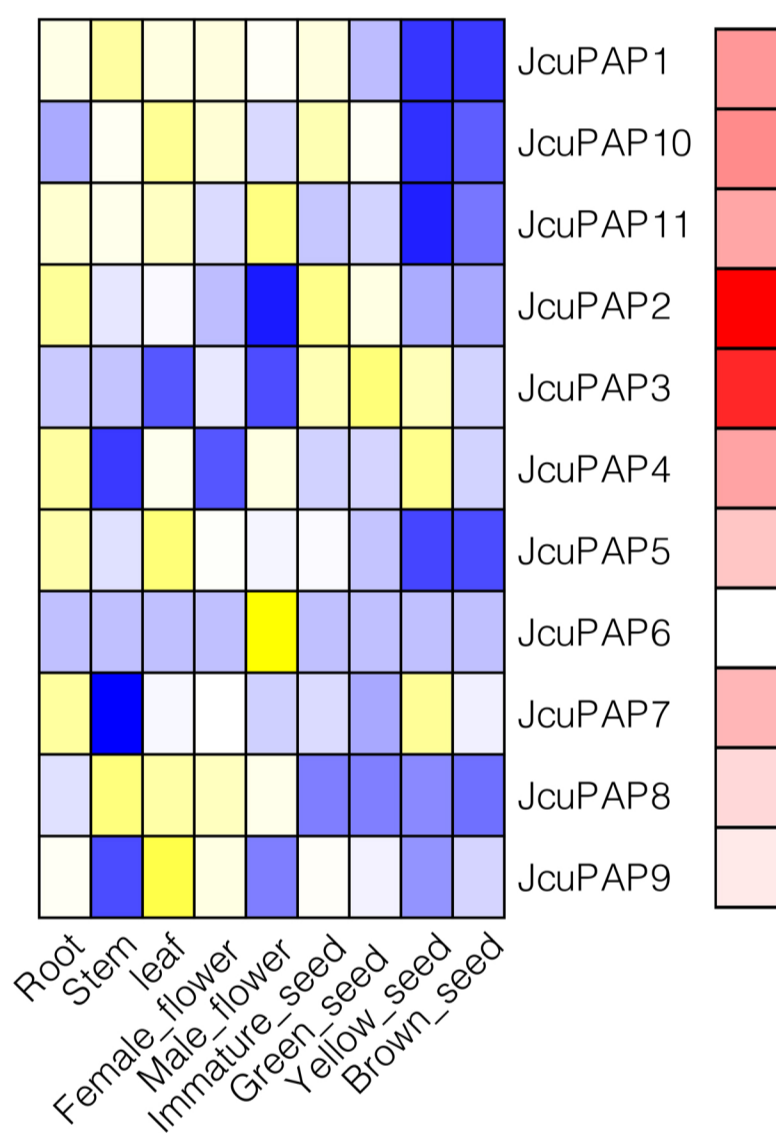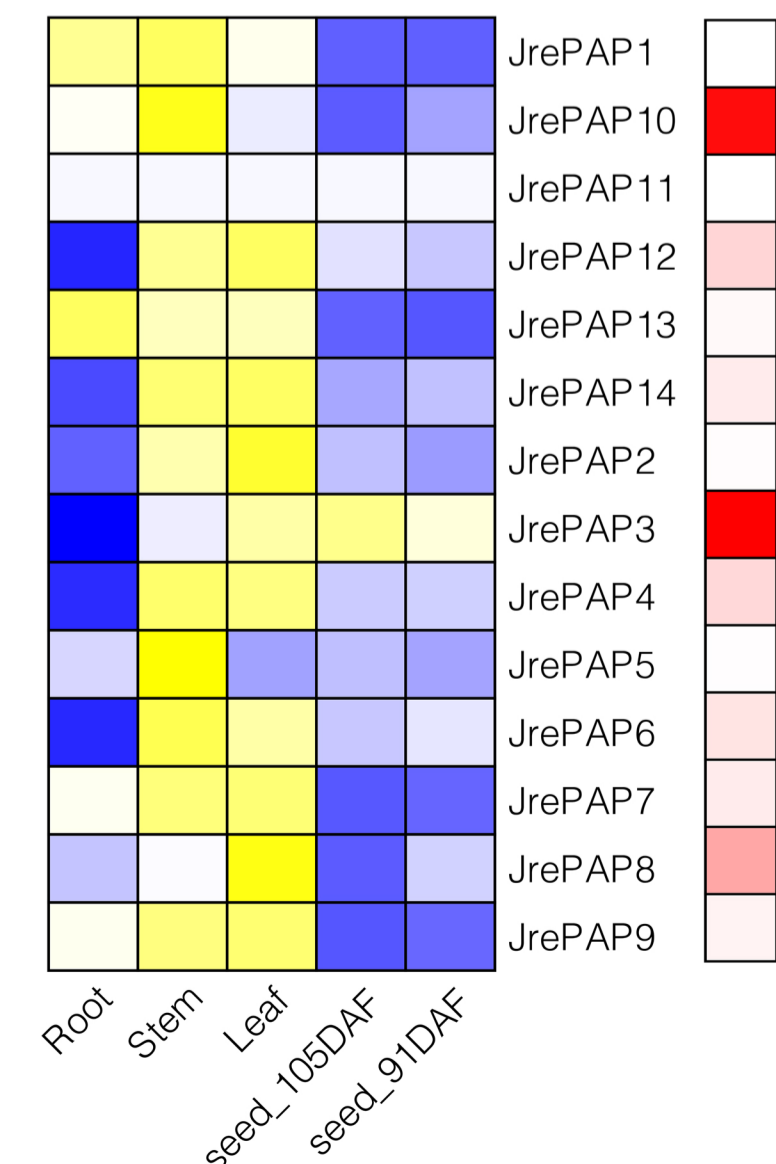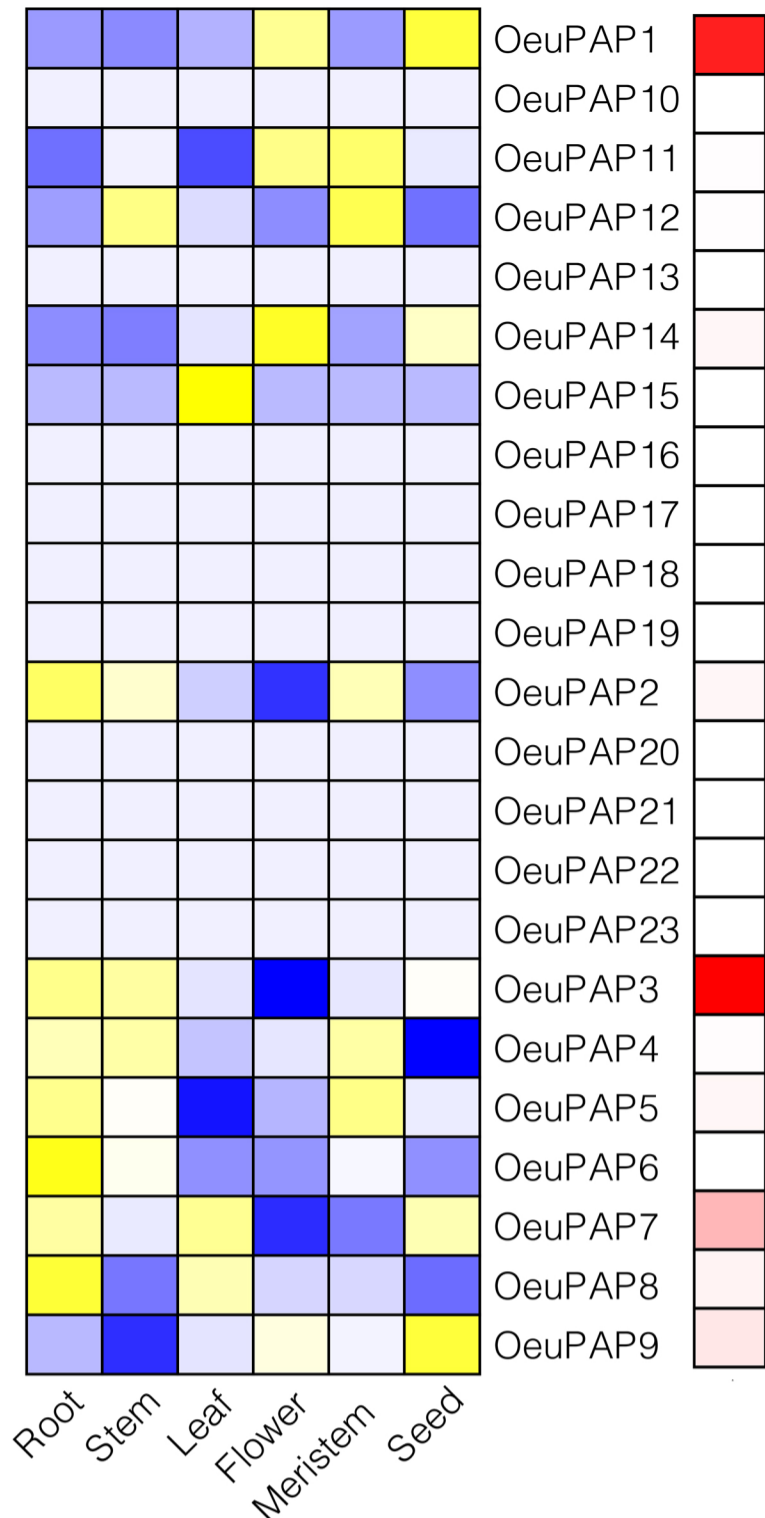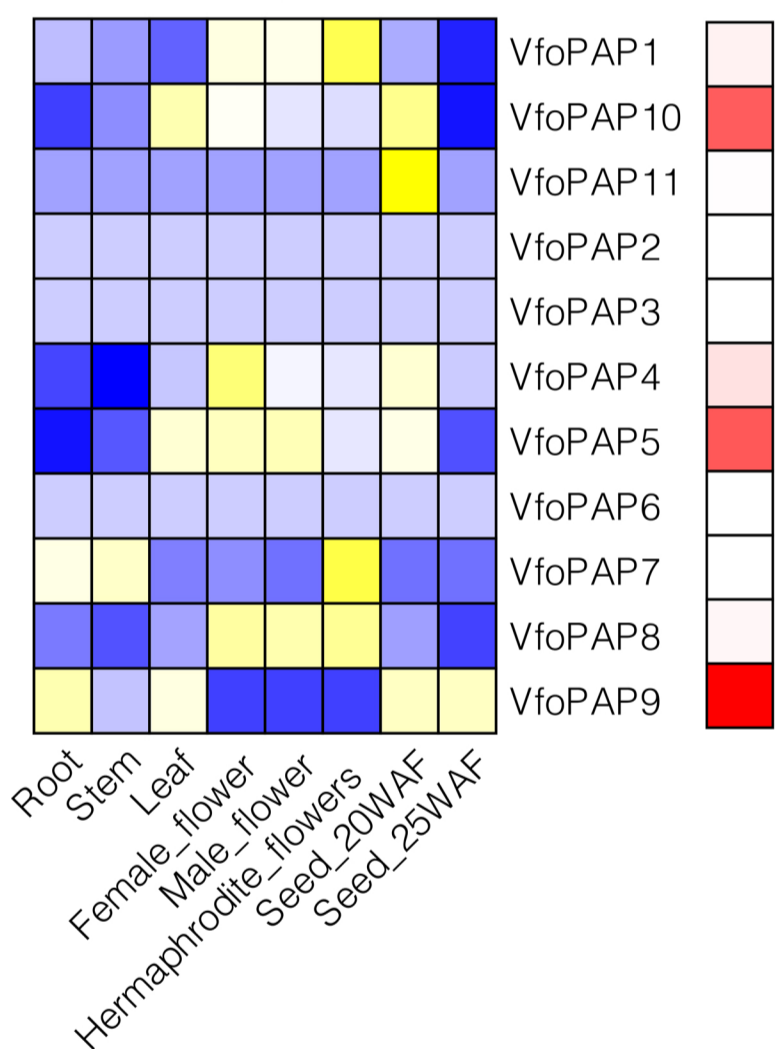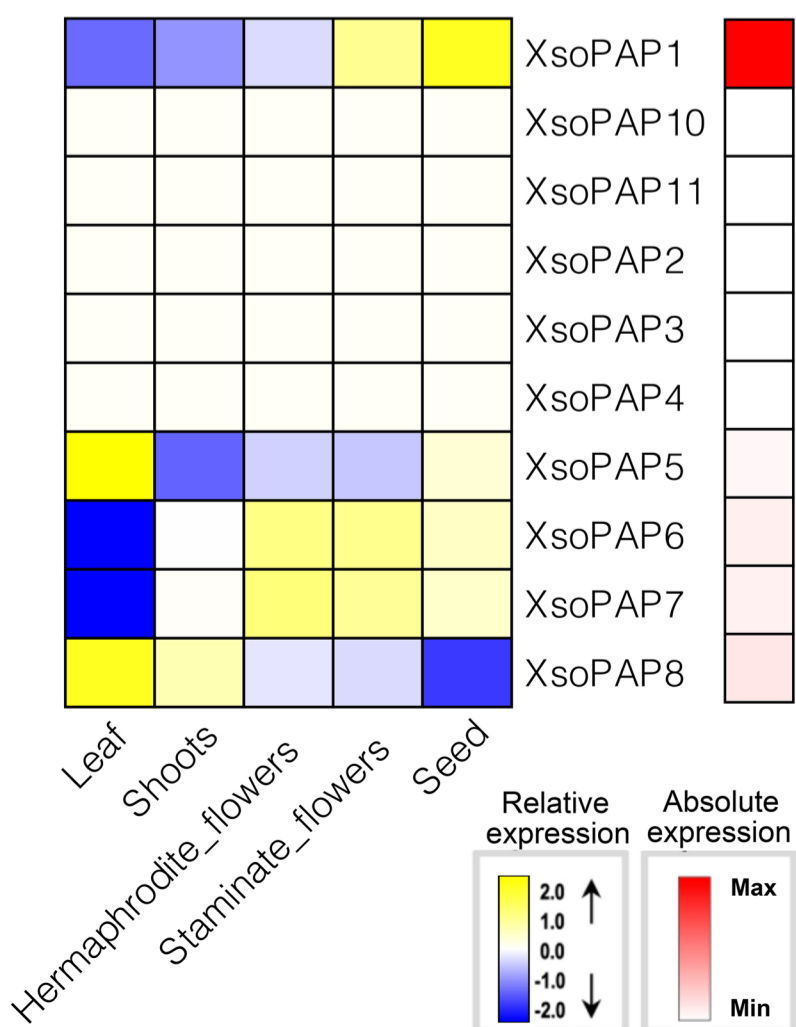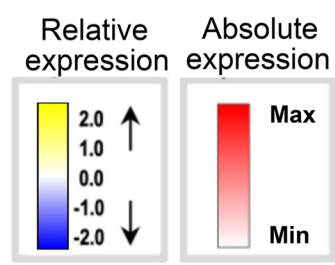

Supplement: Supplementary Figure 2 — The expression profiles of PAP genes in woody oil plants. The expression profiles were obtained by RNA-Seq. Blue and yellow colors indicated low-expression and high-expression, respectively. Red color suggested absolute high-expression. [file DataSheet_2.pdf]
